# Analysis of inter-fraction setup errors and organ motion by daily kilovoltage cone beam computed tomography in intensity modulated radiotherapy of prostate cancer

**DOI:** 10.1186/1748-717X-7-56

**Published:** 2012-04-02

**Authors:** Marcella Palombarini, Stefano Mengoli, Paola Fantazzini, Cecilia Cadioli, Claudio Degli Esposti, Giovanni Piero Frezza

**Affiliations:** 1 Department of Medical Physics, Bellaria Hospital, Bologna, Italy; 2 Department of Management, University of Bologna, Bologna, Italy; 3 Department of Physics, University of Bologna, Viale Berti Pichat 6/2, 40127 Bologna, Italy; 4 Specialization School in Health Physics, University of Bologna, Bologna, Italy; 5 Department of Radiotherapy, Bellaria Hospital, Bologna, Italy

**Keywords:** Prostate, Inter-fraction, ConeBeam CT, Image-guided radiotherapy, Organ motion, Adaptive radiation therapy, Bootstrap statistical analysis

## Abstract

**Background:**

Intensity-modulated radiotherapy (IMRT) enables a better conformality to the target while sparing the surrounding normal tissues and potentially allows to increase the dose to the target, if this is precisely and accurately determined. The goal of this work is to determine inter-fraction setup errors and prostate motion in IMRT for localized prostate cancer, guided by daily kilovoltage cone beam computed tomography (kVCBCT).

**Methods:**

Systematic and random components of the shifts were retrospectively evaluated by comparing two matching modalities (automatic bone and manual soft-tissue) between each of the 641 daily kVCBCTs (18 patients) and the planning kVCT. A simulated Adaptive Radiation Therapy (ART) protocol using the average of the first 5 kVCBCTs was tested by non-parametric bootstrapping procedure.

**Results:**

Shifts were < 1 mm in left-right (LR) and in supero-inferior (SI) direction. In antero-posterior (AP) direction systematic prostate motion (2.7 ± 0.7 mm) gave the major contribution to the variability of results; the averages of the absolute total shifts were significantly larger in anterior (6.3 ± 0.2 mm) than in posterior (3.9 mm ± 0.2 mm) direction. The ART protocol would reduce margins in LR, SI and anterior but not in posterior direction.

**Conclusions:**

The online soft-tissue correction based on daily kVCBCT during IMRT of prostate cancer is fast and efficient. The large random movements of prostate respect to bony anatomy, especially in the AP direction, where anisotropic margins are needed, suggest that daily kVCBCT is at the present time preferable for high dose and high gradients IMRT prostate treatments.

## Background

Intensity-modulated radiation therapy (IMRT) for prostate cancer enables creating a steep dose gradient between prostate and rectum, allowing in principle higher doses to the target and high cure rates while reducing late rectal toxicity [1,2]. Different filling conditions of bladder and rectum can significantly influence the inter-fraction position of the prostate during IMRT [3,4], with consequent modifications of dose distribution in the target and adjacent organs. This may impair local control, with increased risk of late sequelae. To reduce the extra margin needed to allow for prostate motion, an accurate localization of the prostate position at the time of treatment is therefore needed.

Kilovoltage cone beam computed tomography (kVCBCT) is one method to assess and correct for inter-fraction prostate localization immediately before treatment [5]. It enables direct visualization of soft-tissue targets and organs at risk, and with a flat-panel imager may combine volumetric and radiographic/fluoroscopic imaging using the same device. However, daily image-guidance affects the traditional treatment workflow and can increase the patient's radiation dose [6]. Several studies have been published on direct visualization of the prostate observed with different modalities of image guidance, including implanted fiducial markers [7], ultrasound [8], kV or MV computed tomography [9-11] and electromagnetic responders [12]. However, studies are scarce on both setup and organ motion using daily kVCBCT.

In our current clinical practice at Bellaria Hospital of Bologna, daily target localization by kVCBCT (CBCT in the following) has been routinely performed since November 2008. The aim of this study is to determine the inter-fraction setup error and the prostate motion relative to the bony anatomy assessed with CBCT in patients treated for prostate cancer with radical radiotherapy. Results of daily corrections of 18 patients are presented. Moreover, an off-line Adaptive Radiation Therapy (ART) image-guided correction protocol is retrospectively simulated to determine whether the number of images performed could be reduced.

## Methods

### Patients

Eighteen prostate cancer patients treated between January and September 2009 with IMRT were retrospectively studied. Patients were staged according to the National Comprehensive Cancer Network risk class assessment [13]. Each of these patients underwent a 3-mm slice spacing planning CT (PCT) scan on a *LightSpeed *CT scanner (*General Electric, UK*). All patients were instructed to empty the rectum and fill the bladder, drinking 500 ml of water 30 min before the PCT and therapy. Planning target volumes (PTVs) were obtained by using three-dimensional (3D) automatic expansions of clinical target volumes (CTVs), applying 8-mm in left-right (LR) and anterior (A), 5-mm in posterior (P) and 8-mm in supero-inferior (SI) directions. Intermediate and high-risk patients were prescribed 76 Gy to the CTV1 (the prostate and the first proximal third of the seminal vesicles) and 50 Gy to the CTV2 (the rest of seminal vesicles) in 38 fractions with Simultaneous Integrated Boost. Low-risk patients were prescribed 74 Gy in 37 fractions soley to the prostate. Written informed consent was obtained from the patients for publication of this report.

### Image-guidance procedure and treatment

Prostate position was assessed before each fraction through CBCT image guidance (*XVI, Elekta, Crawley, UK*). Patients were positioned first by using lasers and skin marks, then a CBCT was acquired. An average of 36 CBCT (29-38) per patient were obtained and used in this analysis (641 CBCTs).

To quantify the setup error we first performed a pelvic bones match ("B-match") of the CBCT on the PCT scan based on a fully automated 3D chamfer algorithm [14]. Then, the matching was manually adjusted by a RT (technologist) to overlap the prostate on the PCT and CBCT scans through a grey-value based soft-tissue matching ("T-match"), assessing the total inter-fraction error (setup + organ motion). Registrations were based on a rigid-body approach. The so-determined corrections were automatically applied to a robotic table with 6 degrees of freedom (*Hexapod Evo, Elekta-Medical Intelligence, Crawley, UK*) before the patient treatment (*Linac Synergy S, Elekta, Crawley, UK*). Values of inter-fraction setup and total positioning displacements were registered for the three principal axes, in left-right (LR) (X), supero-inferior (SI) (Y), and antero-posterior (AP) (Z), and for the three rotation angles (pitch, roll and yaw). The setup translation components X_B_, Y_B_, Z_B _refer to the B-match, and the total positioning translation components X_T_, Y_T_, Z_T _refer to the T-match, respectively.

Motion of the prostate relative to the bony anatomy was defined as the difference between T-match and B-match. Therefore, the prostate inter-fraction movement vector relative to bony anatomy will be identified by the differences X_T_-X_B_, Y_T_-Y_B_, Z_T_-Z_B_. Positive values for X, Y and Z shifts indicate a left, superior and anterior displacements of the isocenter.

### Statistical analysis

Statistical analysis was performed using Statistical Package STATA 9.0 (StataCorp LP). For each patient, the average deviation μ_i _and the standard deviation σ_i _for B-match, T-match and for organ motion have been calculated in each direction. For the entire population of patients, the mean value of μ_i _values (M, group systematic error), the root mean square of the patient σ_i _(σ, random error), and the standard deviation of the patient means (Σ) were computed. The reported error for the means is one standard error. Normality test of each single variable distribution was carried out using Shapiro-Wilk test [15]. In order to test whether patients behaved differently for X, Y, and Z shifts, different statistics were carried out. Using an F-test, Analysis of Variance (ANOVA) was performed to verify that the patient means were statistically different. As ANOVA is based on the strong assumption of normality of the underlining random variable, for sake of robustness a non-parametric test (Kruskal-Wallis) was performed to compare medians of each patient (p-value was determined using a χ^2 ^distribution). The Levene test was carried out to test the equality of variances of each patient through an F-test. All tests were two-sided.

### Simulation of an off-line protocol: bootstrap analysis of the first 5 CBCT

To test the goodness of a first five-days average estimation for correction of each patient instead of a more costly daily on-line procedure, an ART off-line protocol was simulated and compared with a no-correction protocol based only on skin marks alignment and with our daily online correction protocol. Displacements on X, Y, and Z were defined as the differences between daily correction shifts (total positioning translation components) and the average of the first five days patient's shifts. Those values were taken in absolute terms and cumulative distributions of errors were built. Cut-off margins ranging from 1 mm to 8 mm were considered, and the percentages of the observations outside each cut-off level were computed. For each of those cut-offs, confidence intervals were obtained using a non-parametric bootstrap procedure as in Efron, 1979 [16]. Non-parametric bootstrapping is a statistical method that can be implemented by constructing a large number of simulated samples from the original dataset, each of which is obtained by random sampling empirical observations from the original dataset. As a final results, instead of having a single sample, one is able to get a large number of samples, each having the same statistical properties of the previous one as they are derived from it. Meanwhile, the great advantage of bootstrap is to derive estimates of standard errors and confidence intervals for complex estimators of parameters of the distribution, how in the case of the present study.

### Interobserver variability study

T-match method implies a manual intervention of the operator. To evaluate the inter-observer variability, four RTs usually performing the CBCT/PCT matching were asked to match 9 randomly chosen CBCT acquisitions of 3 patients included in this study.

## Results

### Evaluation of systematic and random errors

The systematic rotations of the bones, as well as the rotations of the organ relative to the bones around the three axes were negligible (less than 1.2 degree). For that reason, further analysis has been performed on translations only.

Interobserver variability in T-match method was found to be within 0.9 mm (1 SD) in left-right (LR) (X) direction, and within 1.5 mm and 1.8 mm (1 SD) in supero-inferior (SI) (Y) and antero-posterior (AP) (Z), respectively. Mean values μ_i _and standard deviations σ_i _of total positioning, setup and prostate motion translational shifts registered for all treatment fractions in each direction for the 18 patients are reported in Table 1 along with systematic (M) and random errors (σ) for each variable, and standard deviation of the μ_i _values (Σ). In X and Y directions, M values are both on the order of 1 mm or less. The σ and Σ values in X_T _and X_B _are very similar, indicating a major contribution from bone misalignment, while systematic and random errors calculated for internal organ motion seem to be small in this direction. In Y direction, the setup and the internal organ motion components seem to contribute equally to the total σ and Σ values. It is worth noting the larger values for M, σ and Σ for the total shift in the Z direction (Z_T_) and for the internal organ motion (Z_T_-Z_B_), in comparison with those of the X and Y directions. The basically larger value of M for Z_T _than for Z_B _seems to indicate a significant systematic internal organ motion relative to the bone structure in that direction. Also values of σ and Σ are higher than for X and Y, with a slightly higher contribution from setup errors to the total positioning errors than from internal organ motion. These data are in agreement with setup errors previously reported for prostate cancer patients [10,11], except for the higher inter-fraction prostate motion along the Z direction. Parametric and non-parametric tests show (Table 1) that all the differences are significant. The patients have significantly different means (F test, p-value < 1%), medians (KW, p-value < 1%) and standard deviations (Levene, p-value < 5%) for all variables.

**Table 1:** Statistics of variables (in mm) for each patient (i) and for all fractions for X (LR), Y (SI), Z (AP) directions

	X_T_		X_B_		X_T_- X_B_		Y_T_		Y_B_		Y_T_- Y_B_		Z_T_		Z_B_		Z_T_-Z_B_	
	
Patient:	μ_i_	σ_i_	μ_i_	σ_i_	μ_i_	σ_i_	μ_i_	σ_i_	μ_i_	σ_i_	μ_i_	σ_i_	μ_i_	σ_i_	μ_i_	σ_i_	μ_i_	σ_i_
	
1	4.1	2.4	4.7	2.2	-0.6	1.6	-2.0	2.0	-0.9	1.8	-1.1	1.6	-0.1	3.7	-1.4	2.2	1.3	2.3
	
2	4.2	3.4	4.9	2.7	-0.8	1.3	-3.7	3.6	-1.3	1.5	-2.4	3.5	6.2	4.7	9.6	4.2	-3.4	3.0
	
3	-2.2	2.0	-0.8	1.7	-1.3	1.1	0.5	1.9	0.7	1.4	-0.2	1.9	-4.6	3.1	-4.3	2.9	-0.4	1.2
	
4	-2.1	1.8	-1.4	1.4	-0.8	1.1	0.6	3.0	-0.5	1.9	1.1	2.6	5.9	4.7	-1.1	3.1	7.0	3.0
	
5	1.3	2.0	1.5	2.0	-0.2	0.8	0.6	2.3	0.3	1.5	0.3	1.5	0.7	2.3	-1.3	2.1	1.9	1.7
	
6	-4.0	3.3	-2.4	2.9	-1.6	1.5	0.9	2.2	1.2	1.7	-0.3	1.6	1.3	4.8	-2.1	2.7	3.4	3.9
	
7	0.9	3.2	1.3	2.9	-0.4	0.7	5.0	2.3	4.7	2.1	0.2	1.0	-3.9	4.6	-5.8	2.7	1.9	3.8
	
8	-2.4	2.5	-0.8	1.9	-1.6	1.8	-3.8	3.8	-3.5	1.8	-0.3	3.0	13.4	5.7	5.1	3.6	8.3	4.5
	
9	-3.3	2.8	-2.6	3.0	-0.7	1.1	-4.5	2.6	-0.5	1.7	-4.0	2.7	6.3	4.9	1.7	3.8	4.6	3.2
	
10	0.3	3.5	0.9	3.5	-0.6	0.6	-0.5	1.6	1.5	1.2	-1.9	1.3	4.0	3.1	2.6	2.8	1.4	1.5
	
11	5.3	2.4	6.7	2.5	-1.4	1.2	-4.7	2.8	-1.8	3.0	-2.9	1.7	8.5	4.0	8.9	3.5	-0.4	1.8
	
12	0.3	2.8	0.8	2.6	-0.5	0.8	-2.3	1.9	-1.2	1.3	-1.1	1.8	5.2	3.1	1.0	3.2	4.3	2.2
	
13	-1.0	1.9	0.4	1.3	-1.3	1.0	-1.9	2.7	0.1	1.5	-2.0	2.5	2.2	3.2	1.8	2.2	0.4	2.0
	
14	-0.7	3.0	0.7	2.3	-1.4	1.5	0.1	2.0	1.1	1.4	-1.0	1.5	0.0	4.1	-2.6	2.8	2.5	3.0
	
15	-0.7	1.8	0.7	1.5	-1.4	1.1	0.7	2.1	1.9	2.3	-1.2	1.9	-3.0	5.6	-5.4	5.8	2.4	3.0
	
16	1.8	1.9	3.8	1.4	-2.0	1.1	-1.8	2.5	-2.9	1.7	1.1	1.8	7.9	2.8	2.4	1.8	5.4	2.1
	
17	-5.1	3.4	-4.4	3.2	-0.6	1.0	-0.8	1.8	-0.2	1.8	-0.6	1.5	0.2	3.1	-2.2	3.1	2.4	2.0
	
18	1.6	2.5	2.9	2.0	-1.3	1.3	-2.8	2.1	-0.8	1.4	-1.9	2.0	5.5	4.1	0.6	3.4	4.9	3.3
Errors:	M	σ	M	σ	M	σ	M	σ	M	σ	M	σ	M	σ	M	σ	M	σ
	
	-0.1	2.6	0.9	2.4	-1.0	1.2	-1.1	2.5	-0.1	1.8	-1.0	2.1	3.2	4.1	0.4	3.2	2.7	2.8
	
Σ		2.9		2.9		0.5		2.4		1.9		1.3		4.7		4.3		2.8
	
F *	39.9		50.5		6.2		34.5		42		15.8		48.8		62.5		36.7	
	
KW-χ^2 ^*	359.9		399.9		98.9		314.5		317.6		206.5		361.9		383.4		330.1	
	
Levene *		2.2	.	2.6		4.5		2.7		3.5		4.5		3.6		5.2		5.1

*The values shown in the Table for the statistical tests F, KW-χ^2 ^show that all the differences are significant with *p *< 1% and the Levene test with *p *< 5%

**Figure 1:**
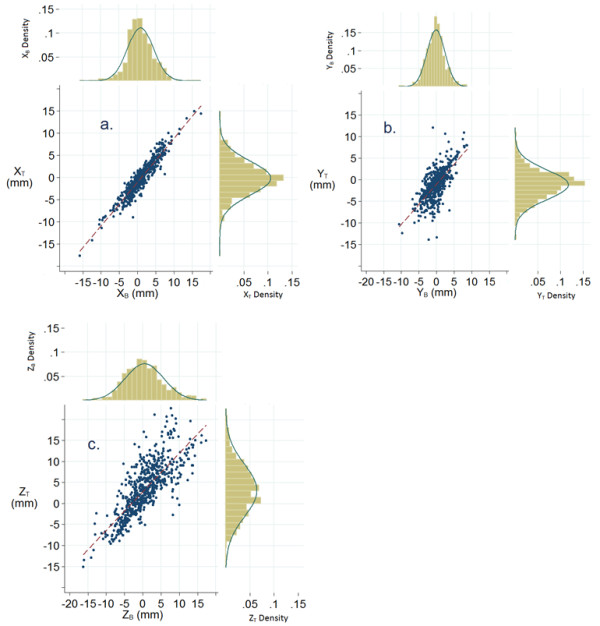
**Histograms of the shifts along × (LR), Y (SI), Z (AP) for the two matching modalities and corresponding scatterplots**. Distributions of the shifts (in mm) along left-right (X) (a), supero-inferior (Y) (b), and antero-posterior (Z) (c) axes for the two matching modalities (B-match and T-match), along with the scatterplots of the corresponding shifts. Bell curves are computed using mean and standard deviation of the underlining variables. Dashed lines represent the bisectors. The scatterplots shown in Figure 1a (X_T _*vs *X_B_), Figure 1b (Y_T _*vs *Y_B_), and Figure 1c (Z_T _*vs *Z_B_), have correlation coefficients of 0.94, 0.74 and 0.78, respectively, all highly statistically significant (*p*-value < 0.001). Note that Z_T _tends to be larger than Z_B_, especially for larger values of Z_B _(Figure 1c).

Figure 1 (a,b,c) shows the histograms of the shifts in the three directions, relative to the PCT scan, registered in the two modalities of matching (X_B_, X_T_; Y_B_, Y_T_; Z_B_, Z_T_) and the scatterplots of the couples of corresponding shifts, for the aggregate set of data (all patients, all fractions). The histograms allow us a rough evaluation of the shift distributions and to compare the two matching modalities. The null hypothesis of normality of the distribution is rejected in all cases (p-value < 1%), due to the high values of the kurtosis, in turn due to the substantial tails on both sides of the distributions. On the other hand, all the distributions are symmetric (skewness close to 0), allowing one to consider the mean and the standard deviation of each set of the data as a good parameter to represent each distribution. The correlation matrix computed on all data between the three translation shifts acquired in the two matching modalities shows that the highest correlation coefficients are observed for the couples of shifts along the same axis (Figure 1).

### Absolute shifts and evaluation of margins

**Figure 2:**
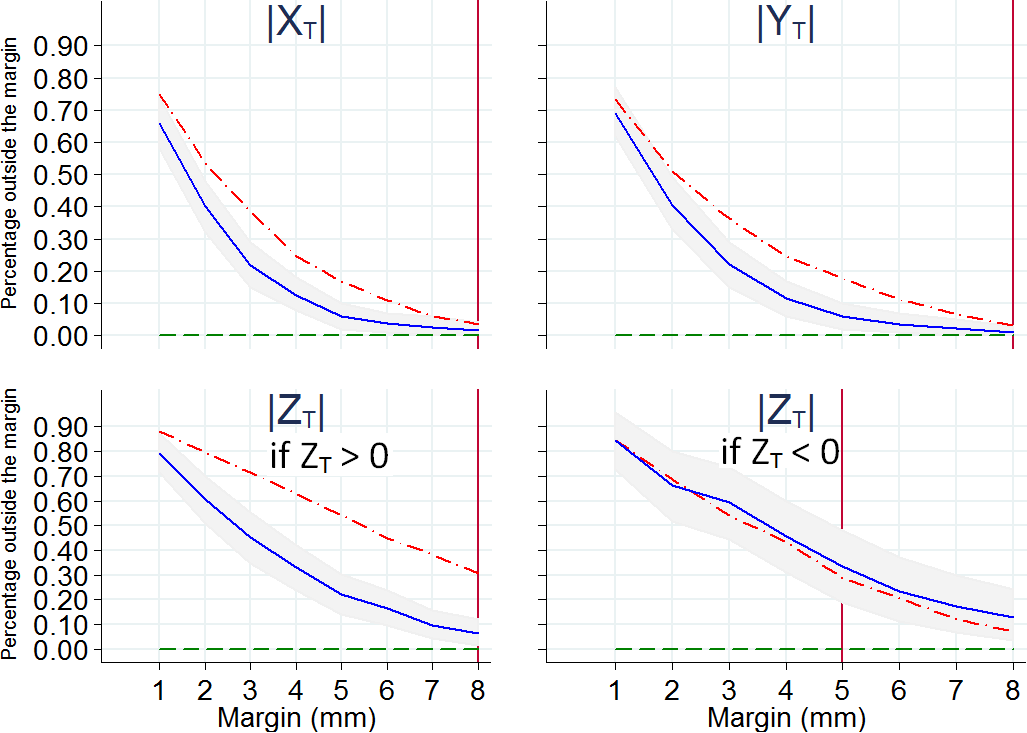
**Percentage of the total fractions outside the margins, for margins from 1 to 8 mm**. The data (all the fractions, all the patients) are computed by using the absolute values of the shifts given by the T-match. Dotted-dashed lines show the results if no correction is made on patient position. Solid lines show the results of a simulated off-line protocol based on the first 5 CBCTs. The area in grey represent the 5% and 95% confidence levels. Dashed lines show the results if a daily correction is made on the basis of daily CBCT. For the antero-posterior (Z) axis the percentages are divided into two cases, Z positive and Z negative, because there is no symmetry in the margins we apply for the positive and negative directions. The vertical lines are positioned at the margins currently used.

The absolute values of the shifts determined by the T-match (X_T_, Y_T_, Z_T_) allow us to evaluate the percentage of fractions for which the absolute values would exceed the margin by different protocols. Figure 2 shows the results for X, Y, Z.

In X and Y directions the percentage of treatments that would be outside the margins of 8 mm is less than 5%. By assuming, for example, a margin of 5 mm, about 20% of the treatments would result outside the margin along X and Y axes, in both positive and negative directions.

Along Z two cases have been considered: Z_T _> 0 and Z_T _< 0 (anterior direction, 446 observations, mean of the absolute values 6.3 ± 0.2 mm; posterior direction, 192 observations mean of the absolute values 3.9 ± 0.2 mm; statistically different p < < 1%). Only for three observations the value was zero and were not considered. With a margin of 8 mm in the anterior direction, about 30% of the treatments would result outside the margin. With margins of 5 mm this percentage would grow to about 55%. In the posterior the choice of 5 mm margin would have left outside about 30% of the treatments. Even an hypothetical margin of 8 mm (too deep in rectum to be applicable) would still leave about 10% of the treatments outside the margin.

### Simulation of an off-line protocol: bootstrap analysis of the first 5 CBCTs

The solid lines in Figure 2 simulate the results of an off-line protocol with 5 CBCTs at the beginning of the treatment. In X and Y, by assuming for example, 5 mm margin, the percentage of fractions outside the margins would be reduced from 20% to less than 10% if the mean of the first 5 CBCTs was assumed for each patient and the corresponding average shifts applied for the following fractions. Along the positive direction of Z (Z_T _> 0), by assuming a margin of 8 mm, the percentage would be reduced from 30% to about 5%. In the posterior direction, a 5 mm margin still would leave about 30% of the treatments outside. There would be no improvement in the Z negative direction by using the average of the first 5 CBCTs.

## Discussion

The extensive statistical analysis of the corrections to make to the position of the patient in the image-guided radiation therapy (IGRT) of prostate cancer after daily kilovoltage CBCT allowed us to get information on the correctness of our protocol, on the organ movement relative to bony anatomy, on the large variability of patients and on the use of an ART protocol.

The systematic and random errors of setup and organ motion along LR (X) and SI (Y) for each patient are of the order of a few mm. This demonstrates the correctness of our positioning procedure. Both parametric and non-parametric statistical tests show that patients behave differently from each others.

In AP (Z) direction, it is the organ motion that gives the major contribution to the variability, both intra and inter-patient. This axis is the more critical, probably due to the variability in patient's rectal distension, although we attempted to treat each patient with an empty rectum. Our findings are in agreement with other reported studies showing that variable rectal filling is the major cause of movement of prostate and seminal vesicles [3,4].

Table 1 confirms that along X axis, the major contribution to the total positioning error is due to bone misalignment, while along Y and Z bone and organ motion shifts are of the same order, with a larger organ motion for Z than for X and Y.

The statistics of the absolute values of Z_T _for positive and negative values confirm the utility of different margins in antero-posterior directions, with the anterior one larger than the posterior one. This is probably one of the major findings of this study.

More than 95% of the prostate displacements in X and Y direction would be within the 8 mm-margins currently used in our center, even in absence of any correction, as shown in Figure 2 (dashed-dotted curves).

Conversely, in Z direction, only 70% of the displacements were within the currently used margins. As a consequence, our findings seem to indicate that, despite setting a daily patient alignment on skin marks, large displacements could occur. Facilities that use this traditional protocol without image-guidance should address strategies to reduce the internal organ motion such as regularly emptying the rectum [11,17] or applying other modalities [18] before the treatment in order to avoid missing the target and/or increased toxicity to organs at risk.

Several ART protocols have been tested to try to reduce the costly daily image guidance procedure, limiting the systematic error Σ but not the random error, σ [19-21].

In this study we compared the percentages of treatments that would exceed the margins if no corrections are made with an ART off-line protocol using the average of the first 5 CBCTs as an offset for all the following fractions of each patient. In X and Y direction, by assuming for example, 5 mm margin, the percentage of fractions outside the margins would be reduced from 20% to less then 10%. Moreover, the curves show that, by applying this off-line protocol, a 6 mm-margin would probably be adequate to compensate for the majority of inter-fraction total displacements.

Again, the Z direction is the most critical. The dashed-dotted lined is outside the area that represent the region contained inside the 5% and 95% confidence levels for X, Y and positive-Z directions. That means that the improvement from 30% to 5% that we would get with this off-line correction protocol is significant, and not obtained by chance. For the negative-Z direction both solid and dotted-dashed lines are contained in the 5%-95% confidence level area. That means that they differ only by chance, and we can consider that there is no improvement in the Z negative direction by using the average of the first 5 CBCTs. This is another major finding of this study.

In the near future, at our centre, an hypofractionated scheme will be introduced for IMRT prostate cancer treatments. As seen in this report, setup and organ motion errors may be relevant, and, if not corrected, they could have a significant detrimental impact on the treatment. For this reason, the actual online daily correction protocol has been maintained for the near future.

The limitations of this study include the fact that uncertainties such as intra-fraction organ motion, contouring delineation of CTVs, intra-observer variability and the intrinsic error caused by the rigid shift approximation were not considered. Further studies will address these issues at our institution.

## Conclusions

The online soft-tissue image-guidance correction based on daily kilovoltage CBCT during intensity-modulated radiotherapy of prostate cancer patients is a fast and efficient procedure. Because of the large random errors that characterize the prostate movement with respect to the bony anatomy, especially in the antero-posterior (Z) direction, we showed that an off-line protocol based on a few CBCTs could improve the treatment margins in left-right (X), supero-inferior (Y) and positive Z direction, but not in negative Z direction with respect to no correction. At present it seems that the daily CBCT is preferable for high dose and high gradients intensity-modulated radiotherapy treatments. Further studies are needed in order to optimize the number of first CBCT scans to be used for an off-line ART procedure.

## Abbreviations

IMRT: Intensity-modulated radiotherapy; kVCBCT, CBCT: Kilovoltage cone beam computed tomography; ART: Adaptive radiation therapy; LR, X: Left-right; SI, Y: Supero-inferior; AP, Z: Antero-posterior; PCT: Planning CT; CTV: Clinical target volume; T-match: Grey-value based soft-tissue matching; B-match: Pelvic bones match; X_B_, Y_B_, Z_B_: Translation components by the B-match; X_T_, Y_T_, Z_T_: Translation components by the T-match.

## Competing interests

The authors declare that they have no competing interests.

## Authors' contributions

MP participated in the design of the study, carried out the treatment plannings, participated in data collection and interpretation, participated in drafting and final revising of the manuscript; SM performed the statistical analysis, including the bootstrap procedure for the ART protocol, participated in data interpretation, in drafting and final revising of the manuscript; PF participated in data interpretation and in drafting and final revising of the manuscript; CC participated in data collection and interpretation, and in drafting the manuscript; CDE participated in the design of the study and in data collection; GPF conceived the study and participated in its design and coordination. All authors read and approved the final manuscript.
